# The Guinea

**Published:** 1892-12-17

**Authors:** 


### PASSING TOPICS.

### THE GUINEA.

One of the most pleasant and versatile of our evening contemporaries has recently given its readers an interesting and instructive homily upon the value of the guinea as a survival of a more picturesque and venerable age than that we are condemned to live in, and incidentally remarks that on a subscription list £1 looks "inexpressibly mean" beside £1 1s. Judging by what goes before, this observation seems intended to apply more particularly to those subscription lists for personal and possibly uncalled for testimonials all too common among us, which are in their essence complimentary, and are not to be compared for a moment with the purely business-like records of assistance rendered to needy and more or less useful and deserving institutions of a charitable character.

In connection with these it must be admitted, we fear, that the aristocratic guinea too often plays a very mean—we had well-nigh written a contemptible—part indeed, and is but a poor and inadequate return for services rendered, which may have cost not a few pounds, although being necessarily unrecorded, the latter are impalpable enough to appear outweighed on the instant by the smallest visible piece of the precious metal.

Ever since we took elementary lessons in mineralogy we have been aware that gold is the most malleable of all metals and that it is among the most tenacious, but we never fully realised its astounding capabilities in these respects until our institutional education began. A guinea, as a reference to charitable sheets will often demonstrate, affords unequalled opportunities for a display of these qualities. By its means we may effectually illustrate Shakespeare's aphorism about keeping the word of promise to the ear and breaking it to the hope. A grain of gold is capable of division into some millions of pieces, each of which, we are told, may be visible to the naked eye, and it is the knowledge of this fact no doubt that induces certain people to multiply their contributions to charity by a process of minute subdivision, which when their "privileges" have been reckoned, leaves the institution in the paradoxical position of being the poorer for their help. For such parsimonious benefactors a simple rule of three sum suffices. If a grain of gold can be made to cover a space of some fifty square inches, over how many years of philanthropic life is it possible to spread one, five, or ten guineas?

Happily, not all souls are self-vexed with this problem; with some the question is rather, how many golden deeds can I compass before my time runs out? Every good cause can count a few such friends as these; every institution depends upon them, and even if they do not tell out all their gifts in dainty guineas, the pounds, so far from looking mean, are an adequate and sufficiently acceptable indication of the wealth and reality of the sympathy which has begotten them.

Agricola.

